# Electronic Phenotype for Advanced Chronic Kidney Disease in a Veteran Health Care System Clinical Database: Systems-Based Strategy for Model Development and Evaluation

**DOI:** 10.2196/43384

**Published:** 2023-07-24

**Authors:** Gajapathiraju Chamarthi, Tatiana Orozco, Popy Shell, Devin Fu, Jennifer Hale-Gallardo, Huanguang Jia, Ashutosh M Shukla

**Affiliations:** 1 Division of Nephrology, Hypertension and Transplantation University of Florida Gainesville, FL United States; 2 Advanced Chronic Kidney Disease and Home Dialysis Program North Florida/South Georgia Veteran Healthcare System Gainesville, FL United States

**Keywords:** advanced chronic kidney disease, EHR phenotype, Veteran Health System, CKD cohort, kidney disease, chronic, clinical, database, data, diagnosis, risk, disease

## Abstract

**Background:**

Identifying advanced (stages 4 and 5) chronic kidney disease (CKD) cohorts in clinical databases is complicated and often unreliable. Accurately identifying these patients can allow targeting this population for their specialized clinical and research needs.

**Objective:**

This study was conducted as a system-based strategy to identify all prevalent Veterans with advanced CKD for subsequent enrollment in a clinical trial. We aimed to examine the prevalence and accuracy of conventionally used diagnosis codes and estimated glomerular filtration rate (eGFR)-based phenotypes for advanced CKD in an electronic health record (EHR) database. We sought to develop a pragmatic EHR phenotype capable of improving the real-time identification of advanced CKD cohorts in a regional Veterans health care system.

**Methods:**

Using the Veterans Affairs Informatics and Computing Infrastructure services, we extracted the source cohort of Veterans with advanced CKD based on a combination of the latest eGFR value ≤30 ml·min^–1^·1.73 m^–2^ or existing International Classification of Diseases (ICD)-10 diagnosis codes for advanced CKD (N18.4 and N18.5) in the last 12 months. We estimated the prevalence of advanced CKD using various prior published EHR phenotypes (ie, advanced CKD diagnosis codes, using the latest single eGFR <30 ml·min^–1^·1.73 m^–2^, utilizing two eGFR values) and our operational EHR phenotypes of a high-, intermediate-, and low-risk advanced CKD cohort. We evaluated the accuracy of these phenotypes by examining the likelihood of a sustained reduction of eGFR <30 ml·min^–1^·1.73 m^–2^ over a 6-month follow-up period.

**Results:**

Of the 133,756 active Veteran enrollees at North Florida/South Georgia Veterans Health System (NF/SG VHS), we identified a source cohort of 1759 Veterans with advanced nondialysis CKD. Among these, 1102 (62.9%) Veterans had diagnosis codes for advanced CKD; 1391(79.1%) had the index eGFR <30 ml·min^–1^·1.73 m^–2^; and 928 (52.7%), 480 (27.2%), and 315 (17.9%) Veterans had high-, intermediate-, and low-risk advanced CKD, respectively. The prevalence of advanced CKD among Veterans at NF/SG VHS varied between 1% and 1.5% depending on the EHR phenotype. At the 6-month follow-up, the probability of Veterans remaining in the advanced CKD stage was 65.3% in the group defined by the ICD-10 codes and 90% in the groups defined by eGFR values. Based on our phenotype, 94.2% of high-risk, 71% of intermediate-risk, and 16.1% of low-risk groups remained in the advanced CKD category.

**Conclusions:**

While the prevalence of advanced CKD has limited variation between different EHR phenotypes, the accuracy can be improved by utilizing two eGFR values in a stratified manner. We report the development of a pragmatic EHR-based model to identify advanced CKD within a regional Veterans health care system in real time with a tiered approach that allows targeting the needs of the groups at risk of progression to end-stage kidney disease.

## Introduction

Advanced chronic kidney disease (CKD) progressing to end-stage kidney disease (ESKD) is a huge burden for the US health care system [1]. Patients with advanced CKD are at increased risk for adverse outcomes, including progression to ESKD and death. Prior studies show that providing pre-ESKD nephrology care and comprehensive pre-ESKD education improves clinical outcomes; reduces health care costs; and increases home dialysis, transplantation utilization, and patient survival [2-6]. Despite these positive outcomes, approximately 40% of patients with incident ESKD in the United States have either limited (less than 6 months) or no access to nephrology care before initiating dialysis and even fewer (<1%) receive kidney disease education services [7,8]. Accurately identifying the advanced (stages 4 and 5) CKD population at risk for ESKD can facilitate targeted needs assessment studies to improve pre-ESKD nephrology care and provide comprehensive pre-ESKD education for this high-risk population [9].

Clinically, CKD is diagnosed by sustained alterations in the structure or function of the kidney for more than 3 months with implications for health. The Kidney Disease: Improving Global Outcomes (KDIGO) Work Group recommends staging CKD based on cause, estimated glomerular filtration rate (eGFR), and albuminuria [10]. Unfortunately, the asymptomatic nature of CKD creates a lack of awareness for patients and providers alike [1,11]. Investigators conventionally use the International Classification of Diseases (ICD)-based diagnosis codes or electronic health record (EHR)-based phenotypes according to the eGFR to identify patients with CKD in clinical databases [12]. These phenotypes recommend using two eGFR values below 60 ml·min^–1^·1.73 m^–2^, obtained more than 90 days apart, to identify a population with CKD of stage 3 or higher in the databases [12]. However, similar guidance is not available to identify an advanced CKD population within clinical databases, and epidemiological investigations frequently use a single latest eGFR value while ascertaining the advanced CKD burden within the database [3,13,14]. Considering the variability in the frequency of measurement, pragmatic fluctuations in the serum creatinine value and concerns for intervening acute kidney injury (AKI) episodes can cause errors in classifying one’s CKD stage [15]. Thus, there is a need to establish an optimal EHR-based method capable of identifying patients with advanced CKD within clinical databases in real time to improve kidney disease care and research.

Using the clinical database of the North Florida/ South Georgia (NF/SG) Veterans Health System (VHS), we sought to assess the burden of advanced CKD prevalence in real time using various EHR-recorded advanced CKD phenotypes within the Veterans Health Administration (VHA) [14,16]. We further examined the accuracy of different EHR phenotypes for advanced CKD by prospectively following the cohorts for 6 months and assessed the number of Veterans remaining in the advanced CKD stage after the initial classification. Furthermore, considering the lack of consensus on EHR phenotyping for identifying an advanced CKD cohort within clinical databases, we also sought to explore a new tiered pragmatic method for estimating the Veteran cohort with advanced CKD in real time.

## Methods

### Data Source and Cohort Selection

**Figure 1:**
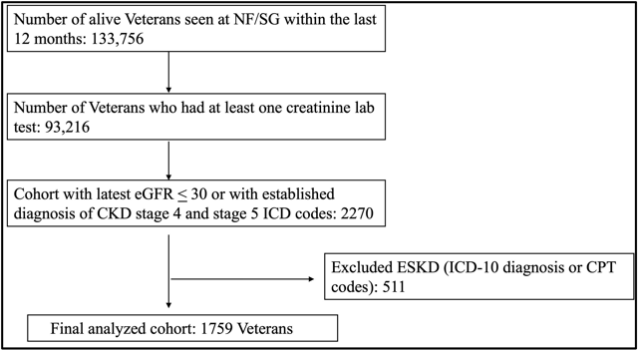
Selection of an advanced nondialysis chronic kidney disease (CKD) cohort at North Florida/South Georgia (NF/SG) Veterans Health System. CPT: Current Procedural Terminology; eGFR: estimated glomerular filtration rate; ESKD: end-stage kidney disease; ICD-10: International Classification of Diseases, Tenth Revision.

This study was conducted as a system-based strategy to identify all prevalent Veterans with advanced (stages 4 and 5) nondialysis CKD. The identified participants were then approached for enrollment in the Trial to Evaluate and Assess the effects of Comprehensive pre-ESKD education on Home dialysis among Veterans (TEACH-VET), which aims to assess the impact of a universal approach for comprehensive pre-ESKD education for all patients with advanced CKD on various clinical, patient-reported, and health services outcomes [17]. We used the Veterans Affairs (VA) Corporate Data Warehouse (CDW) and VA Informatics and Computing Infrastructure (VINCI) to identify the advanced CKD cohort. In brief, the VINCI services initially queried the VA CDW in April 2021 to identify all Veterans registered for service at NF/SG VHS during the 12 months prior to the data extraction (source cohort). The Veterans with an active laboratory value of creatinine were identified and their eGFR was calculated by applying the Modification of Diet in Renal Disease (MDRD) equation [18]. The use of the MDRD equation was determined by the then-prevalent method of eGFR estimation for the VINCI services. We then created a source cohort of Veterans with advanced CKD who either had the latest eGFR value ≤30 ml·min^–1^·1.73m^–2^ (index eGFR) or an existing ICD-10 diagnosis code for advanced CKD (ICD-10 codes: N18.4 and N18.5) within the last 12 months (Figure 1). Patients on dialysis were excluded using the ICD-10 and Current Procedural Terminology (CPT) codes for dialysis (see Table S1 in Multimedia Appendix 1). The prevalence of advanced CKD was estimated in real time using various methods, including advanced CKD diagnosis codes or by eGFR phenotypes described in the literature (ie, by ICD-10 advanced CKD diagnosis codes, by using single [index] eGFR < 30 ml·min^–1^·1.73 m^–2^, and by using the two eGFR values 90 days apart with the index eGFR <30 ml·min^–1^·1.73 m^–2^ and 90-day prior eGFR < 60 ml·min^–1^·1.73 m^–2^) [14,16,19]. The cumulative prevalence of CKD was calculated by combining the data extracted over 6 months. Patient-level data included age, sex, race, ethnicity, religion, marital status, Veteran era, and residential zip codes used for defining the rurality by applying Rural-Urban Commuting Area codes. Statistical analyses were performed using R software version 4.0.4 (R Core Team, 2021) [20].

The source cohort (ie, April 2021 cohort) was divided into a high-, intermediate-, and low-risk advanced CKD cohort utilizing the latest (index) eGFR and 90-day prior eGFR and diagnostic codes (Table 1). Patients with both eGFR values below 30 ml·min^–1^·1.73 m^–2^ were considered to have a high risk of advanced CKD, whereas those with one of the two eGFR values less than 30 ml·min^–1^·1.73 m^–2^ but with the other value ≥30 but <60 ml·min^–1^·1.73 m^–2^ were considered to have an intermediate risk of having advanced CKD. The intermediate-risk cohort with an index eGFR below 30 ml·min^–1^·1.73 m^–2^ was further refined by excluding patients diagnosed with AKI within the 90 days prior to their latest eGFR values using ICD-10 codes. Veterans with both eGFR values ≥30 ml·min^–1^·1.73 m^–2^ but with diagnosis codes for advanced CKD were regarded as having a low risk of advanced CKD (Table 1). The source cohort was followed prospectively for 6 consecutive months until September 2021 using similar queries to examine the eGFR laboratory behavior of the patients with advanced CKD.

**Table 1:** Defining parameters for identifying cohorts at high, intermediate, and low risk of advanced chronic kidney disease (CKD).

Cohort			Index eGFR^a^ (ml·min^–1^·m^–2^)		≥90 days prior eGFR (ml·min^–1^·m^–2^)		Additional criteria
High-risk advanced CKD			<30		< 30		None
**Intermediate-risk advanced CKD**							
	Subgroup 1	<30		≥30 and <60		Excluding AKI^b^ using ICD-10^c^ codes (N17)	
	Subgroup 2	≥30 and <60		<30		Patients have ICD-10 codes for stage 4 and 5 CKD (N18.4 and N18.5)	
Low-risk advanced CKD			≥30		≥30		Patients have ICD-10 codes for stage 4 and 5 CKD (N18.4 and N18.5)

^a^eGFR: estimated glomerular filtration rate; index eGFR refers to the latest eGFR at the time of extraction of the cohort.

^b^AKI: acute kidney injury.

^c^ICD-10: International Classification of Diseases, Tenth Revision.

### Outcomes

The primary goal of this study was to assess the prevalence and accuracy of various EHR phenotypes for extraction of an advanced CKD cohort in a clinical database utilizing diagnosis codes and eGFR models (ie, by ICD-10 advanced CKD diagnosis codes, by using single latest [index] eGFR <30 ml·min^–1^·1.73 m^–2^, and by using the two eGFR values 90 days apart, with the index eGFR <30 and 90 days prior eGFR <60) and our tiered EHR phenotype (high, intermediate, and low risk). Considering that nearly one-third of Veterans do not regularly obtain laboratory testing from within the VA, the denominator population for estimating the prevalence of advanced CKD was judged by only including the Veterans with a valid creatinine value measured over the prior 12 months. Considering EHR phenotypes as a standard for identification of patients with advanced CKD, cross-sectional accuracy for identifying patients with advanced CKD using only ICD-10 codes was assessed by comparison with laboratory-based eGFR EHR phenotypes, analyzed by calculating the sensitivity and positive predictive value (PPV). A manual chart review was conducted in a small randomly selected sample to identify errors related to automated advanced nondialysis CKD identification. Prospective accuracy of all EHR phenotypes, including our pragmatic tiered approach of high-, intermediate-, and low-risk advanced CKD cohorts, was assessed by ascertaining the longitudinal follow-up of laboratory values and identifying the likelihood of remaining in the advanced CKD stage at the end of the 6-month follow-up.

### Ethical Approval

The regulatory approvals for the study were obtained from the institutional review board of the University of Florida (201900870). The study data are stored in secured systems at NF/SG VHS as per the institutional guidelines. 

## Results

We identified 133,756 active enrollees with 93,216 enrollees having at least one value of measured creatinine during an outpatient or inpatient visit at NF/SG VHS in the prior 12 months. After excluding the Veterans with ESKD by additional ICD and CPT codes, a source cohort of 1759 Veterans was identified as either having the latest eGFR ≤30 ml·min^–1^·1.73 m^–2^ or an existing ICD-10 diagnosis code for advanced CKD (ICD-10 codes N18.4 and N18.5) within the last 12 months (Figure 1). The overall cohort had a mean age of 75 (SD 11.1) years and consisted of a predominantly male (95.8%) and white (67.8%) population. These Veterans lived approximately 126.3 (SD 229.5) miles from the nephrology service–providing VA center, with rural Veterans constituting a significant proportion (751/1759, 42.7%) of the cohort (Table 2). A manual chart review was performed on 116 records and 13 Veterans with ESKD were identified, yielding an 11.2% error rate for advanced nondialysis CKD identification.

Of the total cohort of 1759 Veterans, only 1102 (62.9%) had diagnosis codes for advanced CKD, whereas 1391 (79.1%) had the latest (index) eGFR <30 ml·min^–1^·1.73 m^–2^. Incorporating two eGFR values where the latest eGFR was <30 ml·min^–1^·1.73 m^–2^ and the 90-day prior eGFR was <60 ml·min^–1^·1.73 m^–2^, we found 1346 Veterans to have advanced CKD. We then categorized 928 (52.7%) as high risk, 480 (27.2%) as intermediate risk, and 315 (17.9%) as low risk of advanced CKD based on the definitions described above (Tables 1 and 2). The mean eGFR for the initial advanced CKD cohort was 26.2 (SD 12.1) ml·min^–1^·1.73 m^–2^. The mean eGFR was 27.7 ml·min^–1^·1.73 m^–2^ in the ICD codes group, while the mean eGFR in the latest (index) eGFR <30 ml·min^–1^·1.73 m^–2^ group was 22 ml·min^–1^·1.73 m^–2^. The mean eGFR was 20.3 (SD 6.6), 27.4 (SD 5.6), and 42.1 (SD 16.6) ml·min·1.73 m^–2^ for the high-, intermediate-, and low-risk advanced CKD groups in the initial source cohort (Table 2). The prevalence of advanced CKD among Veterans at NF/SG VHS varied between 1% and 1.5% based on the phenotype for advanced CKD. Based on our definitions, the prevalence of advanced (high- and intermediate-risk) CKD at NF/SG VHS was approximately 1.5% (Table 3). The cumulative cohort over the 6 months yielded 1840 Veterans with high and intermediate risk (2% cumulative prevalence). The sensitivity of diagnosis codes was only 55%-65% compared to the eGFR phenotypes, and the PPV of ICD-10 diagnosis codes for advanced CKD varied between 55% and 74% (Table 4).

**Figure 2:**
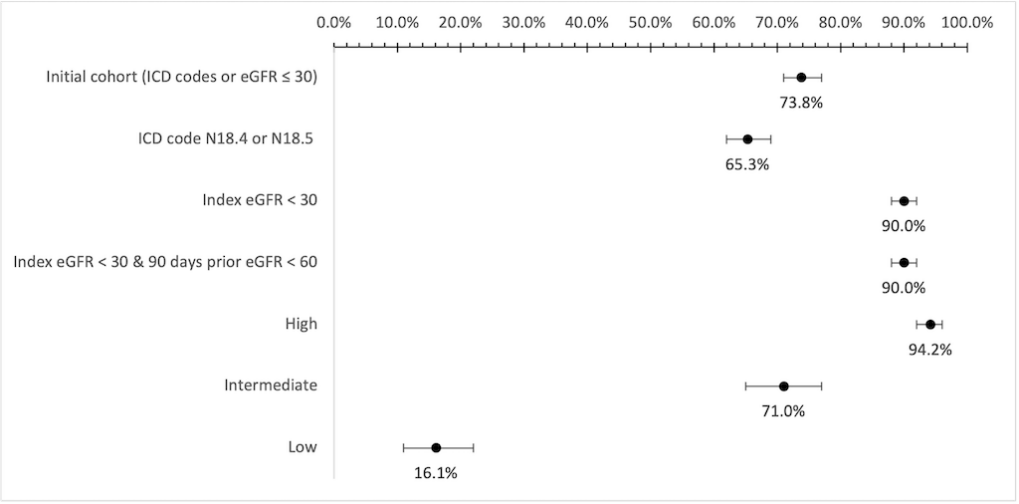
Probability of remaining in advanced CKD stages (4 and 5) based on various EHR phenotypes at 6-month follow-up. CKD: chronic kidney disease; EHR: electronic health record; eGFR: estimated glomerular filtration rate; ICD-10: International Classification of Diseases, Tenth Revision.

The source cohort was followed prospectively for 6 months to examine the variations and likelihood of a sustained reduced eGFR <30 ml·min^–1^·1.73 m^–2^ across various EHR phenotypes. A total of 981 (55.8%) of the 1759 Veterans had at least one subsequent eGFR measurement in the initial April cohort (Table 5). The probability of any subsequent eGFR measurement above 30 ml·min^–1^·1.73 m^–2^ after the index eGFR in the cohort defined by ICD codes was 38.3%, and was approximately 12.7% and 12.8 % in cohorts defined by index eGFR <30 ml·min^–1^·1.73 m^–2^ and two eGFR phenotypes with index eGFR < 30 ml·min^–1^·1.73 m^–2^ and 90-day prior eGFR < 60 ml·min^–1^·1.73 m^–2^, respectively. Similarly, the probability of having any subsequent eGFR value above 30 ml·min^–1^·1.73 m^–2^ after the index eGFR measurement was 7.1%, 35.7%, and 90% in the high-, intermediate-, and low-risk group, respectively. The probability of Veterans remaining in an advanced CKD stage (stages 4 and 5) noted by the recent eGFR <30 ml·min^–1^·1.73 m^–2^ at the end of follow-up was 65.3% in the group identified by the ICD codes, whereas the probability improved to 90% in the group defined by single (index) eGFR <30 ml·min^–1^·1.73 m^–2^ and the group defined by the index eGFR and 90-day prior eGFR method. Similarly, the probability of Veterans remaining in an advanced CKD stage at the end of the follow-up period was 94.2%, 71.0%, and 16.1% for high-, intermediate-, and low-risk groups, respectively (Figure 2, Table 5, and Table S2 in Multimedia Appendix 1).

**Table 2:** Demographic data for the source cohort.

Characteristics		Total cohort (N=1759)	ICD-10^a^ code N18.4 or N18.5 (n=1102)	Index eGFR^b^ <30 (n=1391)	Index eGFR <30 and 90 days prior eGFR <60	High-risk advanced CKD^c^ (n=928)	Intermediate-risk advanced CKD (n=480)	Low-risk advanced CKD (n=315)
eGFR, mean (SD)		26.2 (12.1)	27.7 (13.7)	22.0 (6.5)	22.0 (6.5)	20.3 (6.6)	27.4 (5.6)	42.1 (16.6)
Age (years), mean (SD)		75.3 (11.1)	75.5 (10.9)	75.0 (11.0)	75.2 (10.8)	75.3 (11.0)	75.2 (10.3)	75.5 (12.2)
Sex (male), n (%)		1686 (95.8)	1057 (95.9)	1334 (95.9)	1290 (95.8)	889 (95.8)	461 (96.0)	301 (95.6)
**Race, n (%)**								
	Black	386 (21.9)	232 (21.1)	311 (22.4)	299 (22.2)	216 (23.3)	94 (19.6)	69 (21.9)
	White	1192 (67.8)	758 (68.8)	936 (67.3)	911 (67.7)	616 (66.4)	339 (70.6)	212 (67.3)
	Other or unknown	181 (10.3)	112 (10.2)	144 (10.4)	136 (10.1)	96 (10.3)	47 (9.8)	34 (10.8)
Hispanic ethnicity, n (%)		35 (2.0)	23 (2.1)	31 (2.2)	30 (2.2)	23 (2.5)	9 (1.9)	2 (0.6)
Rural, n (%)		751 (42.7)	475 (43.1)	574 (41.3)	558 (41.5)	378 (40.7)	200 (41.7)	154 (48.9)
Married, n (%)		1087 (61.9)	661 (60.1)	875 (63.0)	846 (63.0)	574 (62.0)	311 (64.9)	179 (57.0)
**Service era, n (%)**								
	Pre-Vietnam	372 (21.1)	244 (22.1)	287 (20.6)	282 (21.0)	207 (22.3)	88 (18.3)	72 (22.9)
	Vietnam	1012 (57.5)	635 (57.6)	803 (57.7)	785 (58.3)	528 (56.9)	292 (60.8)	172 (54.6)
	Post-Vietnam and other	375 (21.3)	223 (20.2)	301 (21.6)	279 (20.7)	193 (20.8)	100 (20.8)	71 (22.5)
Distance to VA^d^ (station 573), mean (SD)		126.3 (229.5)	130.3 (249.0)	127.9 (231.0)	126.6 (225.0)	127.7 (221.2)	124.3 (231.2)	129.7 (258.0)

^a^eGFR: estimated glomerular filtration rate (ml·min^–1^·m^–2^).

^b^ICD-10: International Classification of Diseases, Tenth Revision.

^c^CKD: chronic kidney disease.

^d^VA: Veterans Affairs.

**Table 3:** Prevalence of advanced chronic kidney disease (CKD) based on different criteria.

Prevalence subpopulation definition	Users, n	VA^a^ users with creatinine lab measurement within last 12 months (n=93,216), % (95% CI)	Total VA users (N=133,756), % (95% CI)
Total VA users with at least one creatinine measurement within the last 12 months	93,216	100.0 (100-100)	69.7 (69.4-69.9)
Veterans with ICD-10^b^ code N18.4 or N18.5 within last 12 months	1102	1.2 (1.1-1.3)	0.8 (0.8-0.9)
Veterans with index eGFR^c^ <30	1391	1.5 (1.4-1.6)	1.0 (1.0-1.1)
Veterans with index eGFR <30 and 90 days prior eGFR <60	1346	1.4 (1.4-1.5)	1.0 (1.0-1.1)
Veterans with high risk of advanced CKD	928	1.0 (0.9-1.1)	0.7 (0.6-0.7)
Veterans with high and intermediate risk of advanced CKD	1408	1.5 (1.4-1.6)	1.1 (1.0-1.1)
Cumulative prevalence of advanced CKD (6 months) based on high- and intermediate-risk groups	1840	2.0 (1.9-2.1)	1.4 (1.3-1.4)

^a^VA: Veterans Affairs.

^b^ICD-10: International Classification of Diseases, Tenth Revision.

^c^eGFR: estimated glomerular filtration rate (ml·min^–1^·m^–2^); index eGFR refers to the latest eGFR measurement at the time of extraction of the cohort.

**Table 4:** Diagnostic accuracy of International Classification of Diseases, Tenth Revision codes for advanced chronic kidney disease (CKD) compared to estimated glomerular filtration rate (eGFR)-based defining criteria.

Accuracy metric	Index eGFR^a^ <30 (n=1371), point estimate (95% CI)^b^	Index eGFR <30 and 90 days prior eGFR <60 (n=1346), point estimate (95% CI)	High-risk advanced CKD (n=928), point estimate (95% CI)	High- and intermediate-risk advanced CKD (n=1408), point estimate (95% CI)
Sensitivity	0.55 (0.52-0.57)	0.55 (0.53-0.58)	0.65 (0.62-0.68)	0.57 (0.55-0.60)
Specificity	1.00 (1.00-1.00)	1.00 (1.00-1.00)	0.99 (0.99-1.00)	1.00 (1.00-1.00)
Positive predictive value	0.68 (0.66-0.71)	0.68 (0.65-0.71)	0.55 (0.52-0.58)	0.74 (0.71-0.76)
Negative predictive value	0.99 (0.99-0.99)	0.99 (0.99-0.99)	1.00 (1.00-1.00)	0.99 (0.99-0.99)

^a^Index eGFR refers to the latest eGFR measure (ml·min^–1^·m^–2^) at the time of extraction of the cohort.

^b^20 patients were excluded from this column because they were missing a prior eGFR value; the subsequent column criteria/definitions required two eGFR values, and thus patients without two eGFR values were excluded for consistency between column criteria/definitions.

**Table 5:** Probability of remaining in advanced chronic kidney disease stages (4 and 5) based on various electronic health record phenotypes at the 6-month follow-up.

Characteristic	Initial cohort (ICD=10^a^ codes or eGFR^b^ ≤30) (N=1759)		ICD-10 code N18.4 or N18.5 (n=1102)		Index eGFR^c^ <30 (n=1391)		Index eGFR <30 and 90 days prior eGFR <60 (n=1346)		High risk (n=928)			Intermediate risk (n=480)			Low risk (n=315)	
	Value	95% CI	Value	95% CI	Value	95% CI	Value	95% CI	Value	95% CI	Value		95% CI	Value		95% CI
eGFR, mean (SD)	26.2 (12.1)	26-27	27.7 (13.7)	27-29	22.0 (6.5)	22-22	22.0 (6.5)	22-22	20.3 (6.6)	20-21	27.4 (5.6)		27-28	42.1 (16.6)		40- 44
Days between stage-defining eGFR values, mean (SD)	263.8 (394.3)	245-282	229.0 (304.9)	211- 247	274.3 (399.8)	253-296	260.7 (341.7)	242-279	226.7 (159.3)	216-237	316.0 (526.0)		269-363	296.8 (594.2)		231-363
Subsequent eGFR measurement, n (%)	981 (55.8)	53%-58%	706 (64.1)	61%-67%	753 (54.1)	51%-57%	751 (55.8)	53%-58%	549 (59.2)	56%-62%	238 (49.6)		45%-54%	180 (57.1)		51%-63%
Any subsequent eGFR ≥30 after index eGFR, n (%)	293 (16.7)	15%-19%	271 (24.6)	22%-27%	96 (6.9)	5.7%-8.4%	96 (7.1)	5.8%-8.7%	39 (4.2)	3.0%-5.8%	85 (17.7)		14%-21%	162 (51.4)		46%-57%
Current eGFR ≥30 at 6-month follow-up, n (%)	257 (26.2)	23%-29%	245 (34.7)	31%-38%	75 (10.0)	8.0%-12.0%	75 (10.0)	8.0%-12.0%	32 (5.8)	4.1%-8.2%	69 (29.0)		23%-35%	151 (83.9)		78%-89%

^a^ICD-10: International Classification of Diseases, Tenth Revision.

^b^eGFR: estimated glomerular filtration rate (ml·min^–1^·m^–2^).

^c^Index eGFR: latest eGFR measure at the time of extraction of the cohort.

## Discussion

### Principal Findings

Accurate identification of an advanced CKD cohort within a clinical database can allow large health care organizations to provide targeted evidence-based clinical care, conduct system-wide needs assessment studies, and facilitate clinical and epidemiological outcome studies. Several EHR-based models to identify CKD using ICD codes and laboratory values have been published [12,21,22]. While there is a reasonable consensus regarding the EHR-based strategies to define CKD within a clinical database, no targeted study has examined the feasibility of extracting an advanced CKD cohort in such databases. Exploring the clinical database of one of the largest regional Veterans health care systems in the country, we identified several coding, identification, and accuracy-related concerns in extracting an advanced CKD cohort.

Researchers have conventionally used the provider diagnosis codes to identify and stage patients with CKD in clinical databases. Using the more accurate eGFR-based definitions, several investigators have shown that identifying CKD cohorts purely by diagnostic codes underestimates its true prevalence [23]. For example, Diamantidis et al [24] showed that the clinical recognition of CKD utilizing diagnostic codes was only 11.8% among Medicare beneficiaries. In a systemic review of studies primarily conducted on non-VHA health care databases, Grams et al [23] found that the coding accuracy for CKD varies widely between 8% and 83%, depending on providers’ awareness, and rises with the comorbidity burden and severity of CKD.

Few investigators have evaluated the use and accuracy of CKD diagnosis codes in the VHA clinical database. In a recent analysis of the national VHA database, Saran et al [16] estimated the burden and cost of CKD care on VHA among over 6 million VHA-registered Veterans. While the investigators did not examine the coding accuracy, they found its overall use to be very low (3.2%) compared to much higher estimates (8.02%-27%) obtained using laboratory values [16]. Similar results were recently obtained by Bansal et al [19] in a selective cohort of Veterans with diabetes/hypertension at Veteran Integrated Service Network 17. They found that the laboratory-based prevalence of CKD was approximately 36%, but only 44% of them had diagnosis codes for CKD [19]. Similarly, Norton et al [25] found that 63% of entries lacked CKD codes in a military health system. In conjunction with these reports, our analysis showed that the sensitivity and PPV of diagnosis codes, when compared to the eGFR-based phenotypes, to identify advanced CKD is low, in the range of 55%-65% and 55%-74 %, respectively. Our study further shows that when prospectively followed, nearly one-third of the cohort defined by diagnosis codes had an eGFR value over 30 ml·min^–1^·1.73 m^–2^ at the end of 6-month study. Overall, our findings confirm that the utility and accuracy of diagnosis codes for identifying advanced CKD cohorts in the VHA clinical database is poor.

There are also concerns about using an eGFR-based staging system in clinical databases. EHR-based phenotypes require laboratory measurements of creatinine; however, the regular and periodic availability of creatinine may be inconsistent in the clinical databases. For example, Norton et al [14] showed that only 55% of the study sample had eGFR measurements while validating their CKD EHR phenotype. Similarly, a study examining the VA database showed that only 65% of the VA users had any measurements of eGFR during the study period [16]. This lack of availability of eGFR measures can generate errors in the measurement of disease burden. Further, while the definition of CKD requires the demonstration of a persistent reduction of renal function, many studies report CKD staging statistics using a single eGFR value, with a significant fraction of the cohort lacking the second reported eGFR value. For example, in an analysis performed by the National Kidney Disease Education Program Workgroup, 31% of patients with stage-4 CKD and 36% of patients with stage-5 CKD did not have a prior eGFR <60 ml·min^–1^·1.73 m^–2^ value available [14]. Similarly, in the analysis by Saran et al [16] examining the burden of CKD in the VA database, only approximately 27% of Veterans had two eGFR measurements more than 90 days apart, raising concerns about the accuracy of the disease burden. However, in our analysis, focusing on the advanced stages of CKD, we found that over 1723 (98%) of Veterans had two eGFR values reported for the initial source cohort, substantially increasing the reliability of screening for advanced CKD. Additionally, we noticed that over 55% (n=981) of the source cohort had subsequent measurements of eGFR over the prospective 6 months (Table 5), further providing a more robust overall reliability of our advanced CKD estimates.

While using eGFR-based phenotypes improves the identification of CKD, staging CKD into stages 3, 4, and 5 can be complex in a clinical database due to physiologic variability in creatinine levels, performance of biochemical tests, frequency of measurements, and intercurrent illness and volume status [13]. Examining such variations in repeat estimations over 3-6 months in the VHA database, Shahinian et al [26] reported that nearly 30% of patients with stage-4 CKD and 6% of patients with stage-5 CKD had eGFR values ≥30 ml·min^–1^·1.73 m^–2^ in the repeat measurements, thus misclassifying as advanced CKD instead of CKD stage 3 [26]. These inaccuracies can lead to the misidentification of patients with advanced CKD, creating misappropriations of clinical resource allocation or errors in research outcomes for studies that target a specific advanced CKD population.

Considering these inherent limitations of eGFR and diagnostic codes, we sought to refine the predictive accuracy of isolating an advanced CKD cohort for TEACH-VET by categorizing our EHR-derived source cohort into high-, intermediate-, and low-risk advanced CKD cohorts using the two latest eGFR values obtained 90 days apart. Assessing the cohort prospectively for 6 months, we found a very high and graded level of stability with our tiered approach, with 94% and 71% of Veterans in the high-risk and intermediate-risk groups having a eGFR less than 30 ml·min^–1^·1.73 m^–2^ at the study end point, thus remaining in an advanced CKD stage. These findings suggest that such an operational definition can significantly improve clinical and research decision-making and optimize resource allocations, which is currently used to prioritize and enroll Veterans in a clinical study targeting advanced CKD [17]. At the same time, we show that approximately 16% of those with a low risk for advanced CKD had an eGFR below 30 ml·min^–1^·1.73 m^–2^ at the 6-month follow-up, highlighting the high-risk individuals even among those with apparent inaccuracies in diagnosis codes.

Our study explored various available methods to provide a more optimal method to obtain the population statistics for an advanced CKD burden and stratified this cohort based on their longitudinal probability of requiring stage-specific care. Examining real-time data and accurately determining the denominator to only those with an available eGFR estimation within the prespecified 12-month period, we found that the prevalence of advanced CKD (high and intermediate risk) was 1.5%, which is 2-3 times higher compared to the US general population estimates (0.5%) derived from National Health and Nutrition Examination Survey (NHANES) enrollees [1,27], but is less than VHA estimates (1.62%) provided by Saran et al [16]. Even based on the conservative estimates and accounting for all the VA users as the denominator, the prevalence of advanced CKD seems to be higher than that of the general population (Table 3). Recently, VHA has implemented a clinical tool for identifying a CKD cohort based on a single eGFR measurement [28]. Further refinements in the tool by implementing the proposed tiered risk approach to identify an advanced CKD population can allow the VHA to implement judicious allocation of care and resources to those in the highest need. A manual chart review showed an error rate of 11%, mainly attributed to the Veterans being on dialysis. Although the VA database can be linked to the United States Renal Data System (USRDS) database and help exclude dialysis patients, there is a lag in the USRDS data and hence this might not be helpful when the need for identification of advanced CKD in real time arises, as intended in our study for enrollment into a clinical trial [8,17]. In the VHS system, using the community care dialysis list can further increase the sensitivity of the screened list and reduce the error rate by excluding the Veterans who are currently receiving dialysis.

### Limitations

Our study has a few limitations. In recent times, investigators have described advanced EHR algorithms to identify patients with CKD [29]. However, such phenotypes require complex machine-learning algorithms and validation for the target population, and their application in staging CKD is even further away. This study aimed to explore a pragmatic model for identifying Veterans with high, intermediate, and low risk of advanced CKD in real time that can be easily implemented in routine practice and across a large health care system. Second, we did not incorporate the presence or severity of albuminuria within our parsimonious risk model. However, we believe that it is unlikely to improve upon the model for several reasons. Measurement of albuminuria or even proteinuria is uncommon in clinical databases, including the VHA database, and frequently requires the use of proteinuria categorization on routine urinalysis. The risk for complications and adverse outcomes is significantly high for advanced CKD, as highlighted in the KDIGO classification, irrespective of the degree of albuminuria. Considering the unreliable availability of urine protein measurement, it is likely to be of limited additional value, if any [10]. We acknowledge that the true significance of our parsimonious approach will require studies examining longitudinal clinical outcomes. Third, our eGFR values are based on the creatinine values and utilizing the MDRD equation, according to the then-prevalent practices of the VA CDW at the time of the study. Since the overall intention of the study was to evaluate the methodologies for identifying advanced CKD cohorts within a health care system such as VHA, this is unlikely to change the outcome of the study. Future analyses will need to consider the updated CKD-Epidemiology Collaboration equations incorporating creatinine and cystine values for more accurate staging of CKD. Finally, it needs to be mentioned that our results are applicable only among the active VHA users rather than all VHA-registered Veterans, and thus may misrepresent the true burden of advanced CKD among the entire Veteran population. EHR phenotypes, in general, may exclude people with reduced access to care. 

### Conclusion

We found that the prevalence of advanced CKD at NF/SG VHS is higher than that in the general population as per various EHR phenotypes, including our EHR model. There is significant discordance between coding and laboratory parameters for the identification of advanced CKD, consistent with other studies. EHR phenotypes based on CKD diagnosis codes alone are insufficient for identification of an advanced CKD cohort in a clinical database. We report a simplified and pragmatic EHR-based model to identify advanced CKD within a regional VHS in real time with a tiered approach that allows allocation of resources to the groups requiring immediate attention and are at risk of progression to ESKD. Further testing of this model is needed to determine its broader applicability across the VHA. If validated, similar models can be tested across the non-VHA databases to identify the true burden of advanced CKD and target clinical care in real time.

## Appendix

Multimedia Appendix 1 ICD codes used for deriving the source cohort and stratifying the advanced CKD cohort (Tables S1) and source data for Figure 2 (Table S2).

## Abbreviations

AKI: acute kidney injury
CDW: Corporate Data Warehouse
CKD: chronic kidney disease
CPT: Current Procedural Terminology
eGFR: estimated glomerular filtration rate
EHR: electronic health record
ESKD: end-stage kidney disease
ICD: International Classification of Diseases
KDIGO: Kidney Disease: Improving Global Outcomes
MDRD: Modification of Diet in Renal Disease
NF/SG: North Florida/South Georgia
NHANES: National Health and Nutrition Examination Survey
PPV: positive predictive value
TEACH-VET: Trial to Evaluate and Assess the effects of Comprehensive pre-ESKD education on Home dialysis among Veterans
USRDS: United States Renal Data System
VA: Veterans Affairs
VHA: Veterans Health Administration
VHS: Veterans Health System
VINCI: Veterans Affairs Informatics and Computing Infrastructure

## Data Availability

All data generated or analyzed during this study are included in this published article and its supplementary information files.
