# Supplementary material for: Electronic Phenotype for Advanced Chronic Kidney Disease in a Veteran Health Care System Clinical Database: Systems-Based Strategy for Model Development and Evaluation
Source: Interact J Med Res. 2023 Jul 24;12:e43384. doi: 10.2196/43384 (PMC10411421; doi:10.2196/43384)
Supplement: Multimedia Appendix 1 [file ijmr_v12i1e43384_app1.docx]

**Table S1. ICD Codes used for deriving the source cohort and stratifying the advanced CKD cohort**

| **ICD-10 Code Number** | **ICD-10 Code Description** |
| --- | --- |
| Late stage of CKD |  |
| N18.4 | CKD stage 4 |
| N18.5 | CKD stage 5 |
|  |  |
| **Exclusion codes** |  |
| N18.6 | End stage of kidney disease |
| Dialysis |  |
| 5A1D70Z | Performance of Urinary Filtration, Intermittent, Less than 6 Hours Per Day |
| 5A1D80Z | Performance of Urinary Filtration, Prolonged Intermittent, 6-18 hours Per Day |
| 5A1D90Z | Performance of Urinary Filtration, Continuous, Greater than 18 hours Per Day |
| Z99.2 | Dependence on renal dialysis |
| Z49.31 | Encounter for adequacy testing for hemodialysis |
| N17.0, N17.1, N17.2, N17.8, N17.9 | Acute kidney injury |
| **CPT Code Number** | **CPT Code Description** |
|  |  |
| 90951-90970 | End-Stage Renal Disease Services |
| 90963-90966 | End-Stage Renal Disease Services |
|  |  |
| 90922-90925 | Dialysis |
| 90935-90950 | Dialysis |
| 90971-90999 | Dialysis |
|  |  |

**Table S2: Data for Figure 2.**

| **N** | **EHR Phenotypes for Advanced CKD** | **Percent** | **lower CI** | **upper CI** | **Position** | **graph lower** | **graph upper** |
| --- | --- | --- | --- | --- | --- | --- | --- |
| **724** | **Initial cohort (ICD codes or eGFR ≤ 30)** | **73.8%** | **71%** | **77%** | **6.5** | **2.8%** | **3.2%** |
| **461** | **ICD code N18.4 or N18.5** | **65.3%** | **62%** | **69%** | **5.5** | **3.3%** | **3.7%** |
| **678** | **Index eGFR < 30** | **90.0%** | **88%** | **92%** | **4.5** | **2.0%** | **2.0%** |
| **676** | **Index eGFR < 30 & 90 days prior eGFR < 60** | **90.0%** | **88%** | **92%** | **3.5** | **2.0%** | **2.0%** |
| **517** | **High** | **94.2%** | **92%** | **96%** | **2.5** | **2.2%** | **1.8%** |
| **169** | **Intermediate** | **71.0%** | **65%** | **77%** | **1.5** | **6.0%** | **6.0%** |
| **29** | **Low** | **16.1%** | **11%** | **22%** | **0.5** | **5.1%** | **5.9%** |
|  |  |  |  |  |  |  |  |
